# Appropriateness of maternal referral system and its associated factors in Eastern Ethiopia: a facility-based cross-sectional study

**DOI:** 10.3389/fgwh.2025.1473191

**Published:** 2025-05-16

**Authors:** Betelhem Mengist Sharew, Agumasie Semahegn, Shegaye Yibabie Damtie, Nigus Kassie Worku, Abera Kenay Tura

**Affiliations:** ^1^ Department of Midwifery, College of Medicine and Health Sciences, Dire Dawa University, Dire Dawa, Ethiopia; ^2^ School of Nursing, College of Health and Medical Sciences, Haramaya University, Harar, Ethiopia; ^3^ Centre for Innovative Drug Development and Therapeutic Trials for Africa (CDT-Africa), College of Health Sciences, Addis Ababa University, Addis Ababa, Ethiopia; ^4^ Department of Population Family, and Reproductive Health, School of Public Health, University of Ghana, Accra, Ghana; ^5^ School of Medicine, College of Medicine and Health Sciences, Dire Dawa University, Dire Dawa, Ethiopia; ^6^ Department of Public Health, College of Medicine and Health Sciences, Dire Dawa University, Dire Dawa, Ethiopia; ^7^ Department of Obstetrics and Gynaecology, University Medical Centre Groningen, University of Groningen, Groningen, Netherlands; ^8^ Department of International Public Health, Liverpool School of Tropical Medicine, Liverpool, United Kingdom

**Keywords:** appropriateness, maternal referral system, Eastern Ethiopia, referrals, maternal

## Abstract

**Background:**

Given majority of obstetric complications are often unpredictable, an appropriate maternal referral system is crucial to manage life-threatening obstetric complications and prevent maternal deaths. Although Ethiopia is one of the countries with high maternal deaths, there is a paucity of data on the appropriateness of maternal referrals. The aim of this study was to assess the appropriateness of maternal referrals and its associated factors in eastern Ethiopia.

**Methods:**

A facility-based cross-sectional study was conducted among randomly selected women who were referred to the major referral hospitals during pregnancy, childbirth or the postpartum. Data on maternal conditions and referral related information were collected through review of the medical records using structured checklist. Data were entered into EpiData 3.1 and exported to SPSS 20 for analysis. Bivariable and multivariable logistic regression analyses were fitted to identify factors associated with the appropriateness of referrals using adjusted odds ratio (AOR) along with 95% confidence interval (CI). Significant association was declared at *p* < 0.05.

**Results:**

Of 422 maternal referrals reviewed, only 10.1% (95% CI: 7.1–13.1%) were appropriate. Referrals on working days (AOR = 3.77; 95% CI: 1.29–10.99), which arrived during working time (AOR = 3.64; 95% CI: 1.54–8.61), referred from governmental hospitals (AOR = 5.69; 95% CI: 1.33–24.32) or from private/non-governmental organization facilities (AOR = 2.94; 95% CI: 1.09–7.93), those written on standard referral forms (AOR = 5.52; 95% CI: 1.71–17.85), and which contains referral feedback (AOR = 4.90; 95% CI: 1.93–12.47) were more likely to be appropriate maternal referral.

**Conclusion:**

Only one in ten maternal referrals from public health facilities in eastern Ethiopia were found to be appropriate. Referrals on working days and time, from governmental hospitals, private facilities, standard referral forms used, and those with referral feedback were found to be appropriate. Strengthening referral system through focusing on non-working hours and during weekends as well as co-creating standards forms are essential for making maternal referrals appropriate and effective in reducing maternal deaths.

## Background

The majority of maternal mortality is caused by direct obstetric events such as hemorrhage, hypertensive disorders of pregnancy, obstructed labor, sepsis, eclampsia and complications of abortion (1). Maternal mortality continues to be a problem largely for poor women in low- and middle-income countries (LMICs) (2). The current global maternal mortality ratio (MMR) is 223 per 100,000 live birth that is still far from the goal 2030 (70 per 100,000 live births). The vast majority, 94% of these maternal deaths occur in low and middle-income countries (3). At the global level, in 2020, an estimated 287 000 women globally died from a maternal cause, equivalent to almost 800 maternal deaths every day, and approximately one every two minutes. The Sub-Saharan Africa accounts for 70% of global maternal deaths and 43% of global newborn deaths (4). Ethiopia is among the 16 Sub-Saharan countries with the highest MMR of 412 per 100,000 live births that might associated with low access to health service, low utilization and poor quality of care (5). Given majority of obstetric complications are unpredictable, having a system of maternal transfer (referral) to centers with appropriate care is essential to prevent deaths during pregnancy and childbirth.

Referral is a two-way process by which a healthcare worker/health facility transfers the responsibility of care temporarily or permanently to another where the expected quality of care is better (6). Appropriate referrals are characterized by legible and complete referral forms, the use of structured referral forms, following referral guidelines, good communication, smooth transfer of patients preferably to the closest facility, and timeliness (6–9). Maternity care that requires referral is usually due to complications that necessitate the use of life-saving services, or “*signal* functions” as recommended by the World Health Organization (WHO) that cannot be provided by the referring facility (10) Overall, referrals necessity, referral destination and referral quality are the three distinct attributes of maternal referral appropriateness (11).

Health care services in Ethiopia are delivered through extensive national programs and networks in which the referral system is one area of focus (12). Referrals begin at lower tiers of the primary health care system and continue to higher ones although there can be horizontal referrals between similar-level facilities at the request of patients (13). Referral system implementation, however, has been facing challenges as a result of resource- and management-related constraints (14). The capacity of different tiers of public sector health facilities in Ethiopia to function as emergency obstetric and newborn care (EMOC) facilities is varied, with some being Comprehensive emergency obstetric and newborn care (CEMONC) facilities, while others function as Basic emergency obstetric and newborn care (BEMONC) levels. Given this variation, it is important that an effective referral system is in place to facilitate essential first line management at the first facility a mother attends, and efficient transfer to higher level care facilities when complications may necessitate. A dysfunctional referral system can contribute to a poor program impact on maternal and neonatal mortality outcomes. Referral can only be justified if the referral facility provides a reasonable level quality of care (15).

Inappropriate maternal referral systems in Low and Middle Income countries (LMICs) are due to noncompliance with referral policy (10), poor documentation, lack of well-designed referral forms, healthcare providers burn out because of sluggish bureaucracies, no consultation or communication, poorly coordinated referrals between two facilities and working within inadequate resource at referral facilities (11, 16, 17), weak health information systems to capture referral data, and poor transport arrangements for emergency referrals (18). Qualitative study conducted in Ethiopia showed that the main barriers are grouped into three domains, such as: communication, transportation, and healthcare system. and the most commonly reported barriers were lack of pre-referral communication and feedback, using informal communication, incomplete referral forms, poor ambulance service including misuse of ambulances, lack of skilled healthcare escort and lack of medical equipment at emergency, unnecessary self-referrals, poor referral skills and limited number of health professions (19).

Whilst the importance of maternal referral system in improving maternal survival is well documented, appropriateness of maternal referral system in Ethiopia is not well addressed. Even though there are some studies conducted in Ethiopia most of them were qualitative studies and no single was conducted on magnitude of appropriateness of maternal referral system in Ethiopia including study area. Therefore, this study was conducted to assess the magnitude of appropriateness of maternal referral system and its associated factors in eastern Ethiopia.

## Methods

### Study settings and design

A facility-based cross-sectional study design was employed from July 01–31, 2021 in two main referral hospitals: Hiwot Fana Comprehensive Specialized University Hospital (HFCSUH) and Dil Chora Referral Hospital (DCRH) referral hospitals in Harar and Dire Dawa, Eastern Ethiopia, respectively. Harar has a total population of 205,000 of these urban population comprises of 111,052(54.8%). It is the smallest region in Ethiopia and surrounded by different districts of Oromia region, namely Kombolcha and Jarso in Northern side, Gursum and Babile in Northeast, Fedis in Southeast, Aweday and Haramaya in the west side, respectively.

There are thirty-one health posts, eight health centers, thirty-four private clinics and six Hospitals (two governmental and four private hospitals). Hiwot Fana Specialized University Hospital is one of referral teaching hospital, which was established during the occupation of Ethiopia by Italian soliders (1928–1933). Currently the hospital giving services to about 5.2 million communities around Harar and neighboring regions like, Dire Dawa administrative council, Eastern Hararge and Ethiopia Somali Regional (Human resource management, 2019). In eastern Ethiopia, HFCSUH is a major referral teaching hospital affiliated with Haramaya University that is being provided specialized comprehensive healthcare services the obstetrics ward receives self-referred. Low risk as well as high-risk women referred from the nearby health centers and hospitals. On average 1,772 total admissions and 876 maternal referral was reporting to this hospital from other health centers and hospitals.

Dire Dawa city administration is located in the eastern part of the country located 515 kilometer far away from Addis Ababa. According to 2007 Ethiopian census projection for 2015/16, the total population of Dire Dawa administration is 506,000 (58% residing in urban where as 42% residing at rural). It has 9 urban Kebeles (Smallest unit of Ethiopia) and 38 rural Kebeles and 100% geographic access with primary health care. In terms of distribution of health facilities by type in the administration, there are two public hospital (Dil Chora Referral Hospital and Sabian general hospital), and four private hospitals, five health centers, 15 higher clinics, 12 medium clinics, & 31 Health posts (governmental). Dil Chora referral hospital is used as a teaching hospital and it has different departments, which renders comprehensive health services through different departments. Currently the hospital giving services to about 1 to 1.1 million communities around Dire Dawa and neighboring regions like, Eastern Hararge, Ethiopia Somali regional state and Djibuti. Dil Choral referral Hospital also serves as a referral destination for approximately one million population in Dire Dawa and its surroundings. On average 1,212 total admissions and 558 maternal referrals was reporting to this hospital from other health centers and hospitals.

### Population and sampling

Women who were referred to the obstetric and gynecologic wards of both hospitals during pregnancy, childbirth, or within 42 days of termination of pregnancy were eligible for this study. Referred women's chart which could not hold maternal referral letter was excluded from the study. Sample size was calculated using a single proportion formula by considering the following assumptions: 95% level of significance, 5% margin of error, 50% proportion of an appropriate maternal referral system, and 10% for incomplete or missing files(*n* = 422). Two main referral destination hospitals (Hiwot Fana Specialized hospital and Dil Chora referral hospital) were selected by lottery method among the four referral hospitals in Eastern Ethiopia. Then, the sample was proportionally allocated to the size of each hospital annual maternal referral load. Medical registration book of all referrals to the obstetric ward for the past 12 months was retrieved and used for preparing a sampling frame. Finally, a computer assisted simple random sampling technique was applied to select eligible cases for the study.

### Data collection

Data were collected using a structured checklist adapted from literature (20, 21) and the Minister of Health of Ethiopia Standard Referral Form (6). Data was collected by trained midwives. The tool contains four major sections: sociodemographic conditions, obstetric characteristics, reasons for referral, legibility and completeness of referral information, and health care system. The checklist was pretested on 5% of referrals before one year.

### Variables of the study

#### Dependent variable

Appropriate maternal referral system.

#### Independent variables

Maternal age, residency, gravidity, parity, marital status**,** previous use of ANC service, levels of referring health facility, referral catchment area, referring health facility, referring person, referral form, feedback given, arrival time, arrival day, availability of referral guidelines, availability of focal person for referral and transportation system.

## Measurement variables

**Appropriateness of maternal referral:** The measurement of outcome variables “appropriateness of the maternal referral system” was adopted from Phyo-Referral Score (PRS), which consists (pre-referral communication, pre-referral care, unavoidable/necessary cases and referral letter completeness). Then if all four components of the referral system (pre-referral communication, pre-referral care, unavoidable/necessary cases and referral letter completeness) are met, it considers, as the referral is appropriate. Appropriate maternal referral system: if the sum of the four component of the referral system score is 4 which is donated with “1” (it includes mean completeness of the referral letter ≥75% (complete = 1) and if other corresponding variables are “Yes = 1” (pre-referral care, pre-referral communication, unavoidable case). Inappropriate maternal referral system if the sum of the four components of the referral system score is <4 which is donated with “0” (mean completeness of the referral letter is below 75% and if other corresponding variables are “No = 0”. (Pre-referral care, Pre-referral communication and unavoidable case (22).

**Referral letter completeness:** The mean of the referral letter is above or equal to 75% was categories as “complete referral letter” (21).

**Feedback not given:** If the feedback slip is still attached to the referral paper, or if the referral letter does not have a feedback section (23).

**Pre-referral care:** at least vital signs are recorded and appropriate management is given as needed according to the diagnosis and based on national guidelines.

**Pre-referral communication:** If the referring facility informed the receiving hospital before referral.

**Referral sheet illegible:** If it is difficult to read by two person (by data collectors or one more qualified person) (20).

**Unavoidable case/necessary cases:** Unavoidable case was rated with one midwife and one obstetrician and if disagreement between them third midwife or obstetrician was involved for decision. A woman with high risk indicators who cannot be managed at primary or secondary health facilities (20).

**Vital signs measured:** If at least two of blood pressure, temperature, respiratory rate, pulse rate, or oxygen saturation is documented (20).

## Data quality control

Data quality was ensured during collection, entry and analysis. Before conducting the main study, training was given to supervisors and data collectors. A pretest was carried out on 42 charts (10%) of the sample size before one year and the necessary modifications were made. The principal investigator and supervisors were conducting day-to-day on-site supervision during the whole period of data collection. At the end of each day, the checklist were reviewed and checked for completeness and accuracy by the supervisor and investigator and corrective discussion was undertaken by all the research team members. Two data clerks were recruited for the data entry process.

## Data analysis

Collected data were checked for completeness, entered into Epi Data 3.1 and exported to SPSS 20 for cleaning and analysis. Maternal referral system appropriateness was computed using four main components of maternal referral process such as pre-referral communication, pre-referral care, the referral was unavoidable, and referral letter completeness. Then maternal referral system was labeled as “1 = appropriate” and “0 = inappropriate”. Descriptive statistical analysis was carried out frequencies, proportions, mean and standard deviations. Both bivariable and multivariable logistic regression analysis were used to identify association between dependent and independent variables. Variables which had a *p*-value of <0.2 in the bivariable analysis were taken in to the multivariable analysis to control the possible effects of confounders. The Hosmer-Lemeshow goodness of fit test was fitted to check the model's fitness (0.198). Finally, adjusted odds ratio (AOR) along with 95% confidence interval (CI) was used to declare significance association between maternal referral systems at *p* < 0.05.

## Results

### Characteristics of study participants

Of the total of 422 maternal referral records reviewed, 395 (93.6%) which met the inclusion criteria were included. The mean age of women was 25.86 (±5.73) years, ranging from 15–44. Majority of them were 21–30 years old (73.1%), married (94.4%), and had antenatal care (82.6%). More than half were from health centers (56.5%) and multipara (58.2%). Almost two-thirds of the women were referred during the intrapartum period (64.3%, *n* = 254) and gave birth through spontaneous vaginal delivery (67.4%, *n* = 23) (Table 1).

**Table 1:** Characteristics of women referred to hospitals in Eastern Ethiopia 2021 (*n* = 395).

Variables	Category	*N*	%
Age	<18	45	11.4
	18–35	155	39.2
		134	33.9
		36	9.1
	>35	25	6.3
Residence	Urban	172	43.5
	Rural	223	56.5
Marital status	Married	373	94.4
	Other	22	5.6
Parity	Nulli-para	165	41.8
	Multi-para	230	58.2
ANC visit	Yes	326	82.5
	No	69	17.5
Number of ANC visits (*n* = 326)	<4	247	75.8
	>4	79	24.2
Referral period	Prenatal	122	30.9
	Intra-partum	254	64.3
	Postpartum	19	4.8
Mode of delivery (*n* = 350)	Spontaneous vaginal delivery	236	67.4
	Cesarean section	100	28.6
	Instrumental delivery	11	3.1
	Assisted breech delivery	3	0.9
Birth outcome (*n* = 350)	Alive	295	84.3
	Still birth/IUFD	46	13.1
	Newborn death	6	1.7
	Congenital anomaly fetus	3	0.9
Status of women at discharge	Alive	383	97
	Referred out	2	0.5
	Disappear	10	2.5

### Referral characteristics

**Figure 1:**
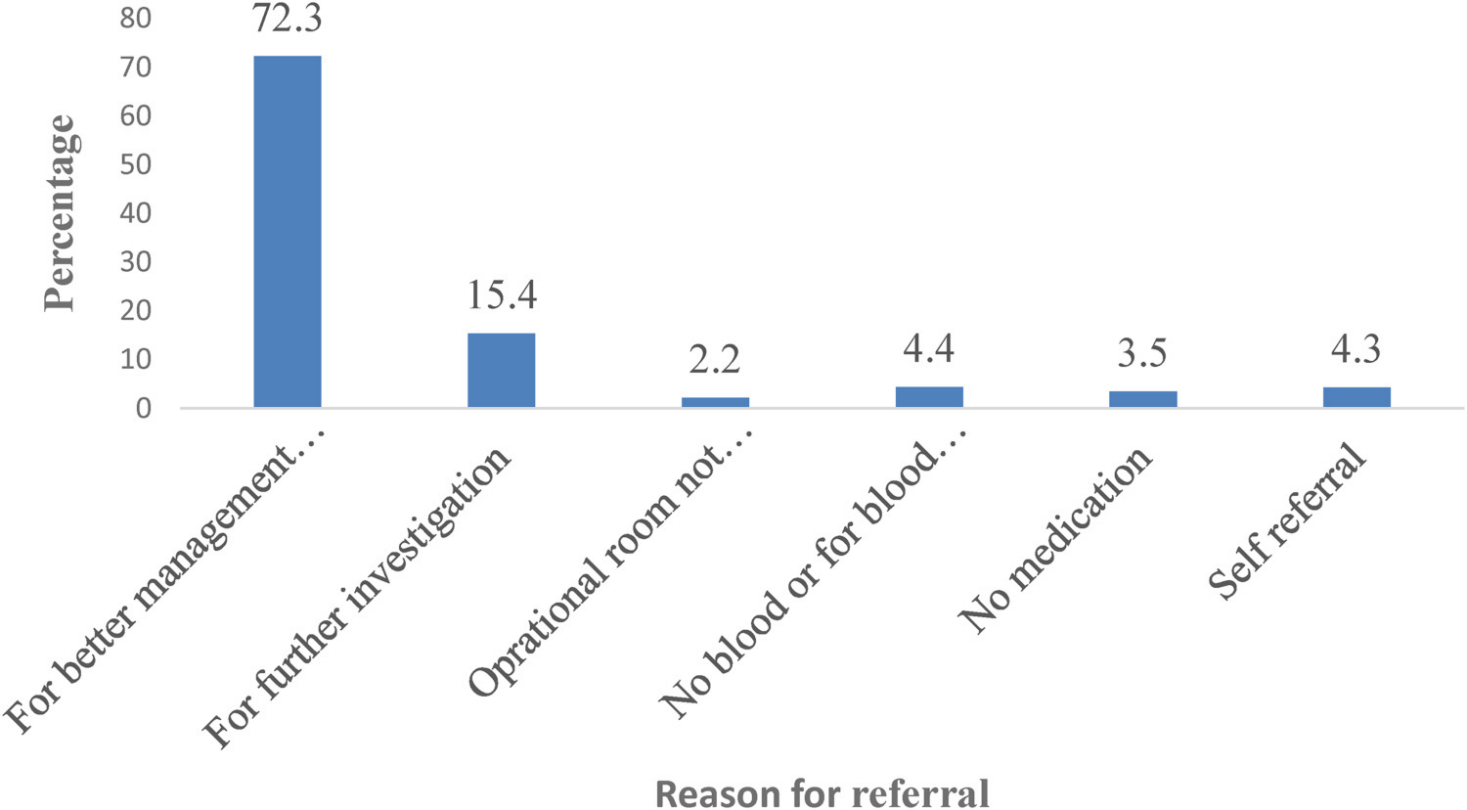
Proportions of reasons for referral among referred women to DCRH and HFSUH, Eastern Ethiopia 2021 (*n* = 318).

Women were referred for some reasons which were beyond the service capacity of the health facility. Seven in ten (70.4%, *n* = 278) referrals were necessary cases or unavoidable cases at the referring health facility (Figure 1).

**Figure 2:**
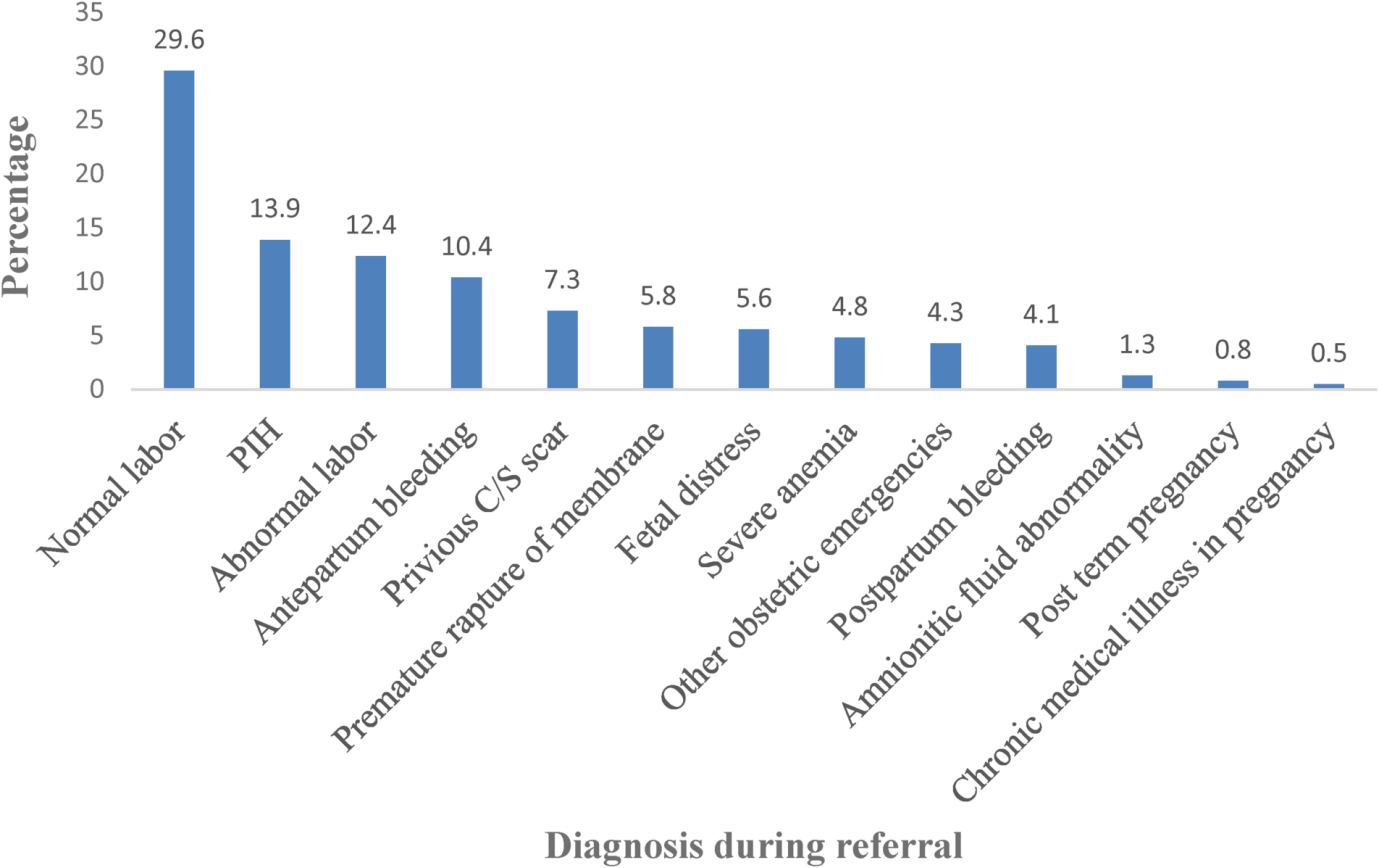
Admission diagnosis for women referred to DCRH and HFSUH, Eastern Ethiopia 2021 (*n* = 395).

The main reasons for referral included antepartum hemorrhage (28.5%) followed by pre-eclampsia (22.2%), and eclampsia (15.3%). Overall, 70.4% of the referrals were unavoidable (Table 2). Despite this, almost all referrals stated a vague reason for referral: *for better investigation and management* (Figure 2).

**Table 2:** Major complications among women referred at referral hospitals in Eastern Ethiopia 2021 (*n* = 145).

Characteristics	*N*	%
Hemorrhagic complications		
Antepartum hemorrhage	41	28.5
Postpartum hemorrhage	15	10.3
Ruptured uterus	5	3.5
Hypovolemic shock	19	13.1
Hypertensive disorders^a^		
Pre-eclampsia	32	22.2
Eclampsia	22	15.3
Gestational hypertension	1	0.7
Aspiration pneumonia	8	5.5
HELLP syndrome 2^0^ to HTD^a^	6	4.1
Other obstetric emergences		
Cord prolapses	4	2.8
Retained placenta	3	2.1
Obstructed labor	8	5.6
Severe anemia	18	12.5
Septic shock	1	0.7

^a^ Multiple responses are possible, HELLP, hemolysis, elevated liver enzyme, low platelets, HTD, and hypertensive disorder.

Three-fourths (74.9%; *n* = 296) of the women reached the referral hospital on a working day, and 54.9% arrived during working time (8:00 AM–5:00 PM). Only 34(8.6%) of the women were referred using the standard referral letter. Moreover, only 54.2% of the referrals contain history of the woman while basic investigations were recorded in 16.7% only. Two hundred eighty five (72.2%) of the referrals cases included a feedback section in their referral forms. But only 191 (48.4%) received written feedback. Overall, 81% (*n* = 320) of the referral forms were incomplete (Table 3).

**Table 3:** Characteristics of maternal referrals in Eastern Ethiopia 2021 (*n* = 395).

Variable	Yes	%	No	%
Legible referral letter	395	93.6	27	6.4
Standard referral form used	34	8.6	361	91.4
Patient identification recorded	365	92.4	30	7.6
Name of referring health facility recorded	375	94.9	20	5.1
Date of referral recorded	354	89.6	41	10.4
Time of referral recorded	48	12.2	347	87.8
Patient history recorded	214	54.2	181	45.8
Vital signs recorded	214	54.2	181	45.8
Diagnosis recorded	367	92.9	28	7.1
Basic investigation recorded	66	16.7	329	83.3
Reason for referral recorded	318	80.5	77	19.5
Management given recorded	143	36.2	252	63.8
Contact number recorded	11	2.8	384	97.8
Name and signature of the referrer recorded	315	79.7	80	20.3
Feedback section included in the letter	285	72.2	110	27.8
Feedback given	191	48.4	204	51.6

Eighty one percent of the referral forms had space for reasons of referral for better management. But 87.7% of the written referral letters submitted with vague reasons for management and investigation' (Figure 2).

### Magnitude of appropriateness maternal referral and associated factors

Of all the referrals, only 10.1% (95% CI: 7.14–13.11) were found to be appropriate. Appropriateness of maternal referral was found to be associated with the referral day, arrival time, types of referring facility, type of referral form used, and feedback practice. Referrals on working days were 3.77 (AOR = 3.77; 95% CI: 1.29–10.99) times more likely to be appropriate compared to those on weekends or holidays. Women who arrived during the working hours (8:00 AM–5:00 PM) were 3.64 (AOR = 3.64; 95% CI: 1.54–8.61) times more likely to have an appropriate referral compared to their counterparts. Compared to women referred from health centers, those coming from public hospitals and private/non-governmental facilities were 6 times (AOR = 5.69; 95% CI: 1.33–24.32) and 3 times (AOR = 2.94; 95% CI: 1.09–7.93), respectively, more likely to have appropriate referrals. Referrals using standard referral forms were 5.52 (AOR = 5.52; 95% CI: 1.71–17.85) times more likely to be appropriate compared to those using other generic papers. Finally, referrals which received feedback were 5 (AOR = 4.90; 95% CI: 1.93–12.47) times more likely be appropriate referrals compared to their counterparts (Table 4).

**Table 4:** Factors associated with appropriate maternal referral system at referral hospitals in Eastern Ethiopia 2021 (*n* = 395).

Variable	Category	Appropriate referral		COR (95% CI)	AOR (95% CI)
		Yes	No		
		*n* (%)	*n* (%)		
Residence	Rural	17 (4.3)	206 (52.2)	1	
	Urban	23 (5.8)	149 (37.7)	1.09 (0.97, 3.62)	1.20 (0.49, 2.46)
Marital status	Other	5 (1.3)	17 (4.3)	1	
	Married	35 (8.9)	338 (85.6)	0.35 (0.12, 1.01)	0.39 (0.11, 1.36)
Referral day	Weekend/holiday	5 (1.3)	94 (23.8)	1	
	Working day	35 (8.9)	261 (66.1)	2.52, (0.96, 6.63)	3.77 (1.29, 10.99)
Arrival time	Non-working time	8 (2.0)	170 (43.0)	1	
	Working time	32 (8.1)	185 (46.8)	3.68 (1.65, 8.20)	3.64 (1.54, 8.61)
ANC visit	No	5 (1.3)	64 (16.2)	1	
	Yes	35 (8.9)	291 (73.7)	1.54 (0.58, 4.08)	1.09 (0.36, 3.30)
Referring facility	Health center	27 (6.8)	320 (81.0)	1	
	Government hospital	5 (1.3)	9 (2.3)	6.58 (2.06,21.04)	5.69 (1.33,24.32)
	Private/NGO clinic	8 (2.0)	26 (6.6)	3.65 (1.51, 8.83)	2.94 (1.09,7.93)
Standard referral form used	No	33 (8.4)	328 (83.0)	1	
	Yes	7 (1.8)	27 (6.8)	2.58 (1.04, 6.37)	5.52 (1.71,17.85)
Feedback given	No	10 (2.5)	194 (49.1)	1	
	Yes	30 (7.6)	161 (40.8)	3.62 (1.72, 7.62)	4.90 (1.93,12.46)

AOR, adjusted odds ratio; CI, confidence interval; COR, crude odds ratio.

## Discussion

This study was conducted to determine the appropriateness of maternal referrals to public health referral hospitals in eastern Ethiopia. In Ethiopia, the appropriateness of referrals between health facilities can be significantly influenced by differences in their levels of care, resources, and expertise. Referring facilities, typically health posts and primary healthcare centers, may lack specialized services and trained personnel to manage complex cases, prompting referrals to receiving facilities, which are usually hospitals equipped with advanced medical technologies and specialists. Additionally, communication and coordination between these facilities are crucial; poor communication can lead to mismanagement of patient care. Geographic accessibility and patient load also play a role, as long distances or overwhelming caseloads at receiving facilities can delay necessary treatment. Furthermore, cultural and socioeconomic factors may influence health-seeking behavior and adherence to referral recommendations, thereby impacting the overall effectiveness of the referral system (24).

We found that only one in ten referrals were appropriate. Referrals on working days, working time, from government hospitals and private facilities, using standard referral forms, and those which received feedback were found to be appropriate. While almost all the referrals were found to be unavoidable, the fact that only a tenth of them are appropriately communicated to the receiving facilities is worrisome as receiving facilities might not knew what was given or not prior to woman's arrival.

Our finding is in line with a mixed study conducted in Indonesia, which showed that 11.1% of the referral cases had good referral quality (22). Conversely, it is much lower than a finding from another study from Indonesia (73%) (25). Given compliance of women and families to referrals is weak in many low resource settings, low appropriateness of referrals would increase non-adherence to referral recommendations.

As expected, off-time and weekend referrals were more likely to less appropriate. This has been echoed in a study from United States of America which has found significant differences between weekend and weekday work environments, like supervision and problems getting (physician) backup for emergencies (19). Weekends and/or holidays activities are carried out by fewer staff so that fewer number of clinicians and less supervision felt serious lapses in patient safety (19). Since fatigue generated by long hours of work before taking a shift in a maternity ward, excessive hours of work, and excessive responsibility, especially at night-time, seem to interfere in decision-making and quality of care provided, relating to possible adverse maternal and perinatal outcomes. This study is supported by the study conducted in Brazil which stated that alertness of health care providers regarding patient care is decreasing during night time (20). Another study found that those night work assignments were similar to those in the daytime, but are carried out by fewer staffs (19). Healthcare providers adequacy is influenced by the time in which nursing care is to be delivered, especially the effects of the healthcare providers work shifts (19).

We found that women who were referred from public hospitals and private facilities more likely to have an appropriate maternal referral system compared to those coming from health centers. This was not unexpected given the cadre of health professionals working in health centers compared to hospitals and private facilities. Almost all health centers are staffed by midwives and/or public officers compared to hospitals and private clinics which are led by obstetricians. Since private hospitals also have the standard for not keeping their patients waiting for long time, timely referral is better than health centers. Appropriate referral system was also more likely among referrals using standard referral forms compared to their counterparts. A cross-sectional study in Kenya that indicated the use of a standardized referral letter that would serve to channel clinical information both upward and downwards in the referral chain is essential for the referral system (22). In addition, a study from Denmark has also clearly described that lack of adequate information at referral letter makes the management at a specialty level difficult, as a result, patients cannot be assured of timely access to services (23). Since structured referral letter has improved the quality and standard of referral letters and save time for all healthcare providers, it decrease the delay to refer the patient (24). On the other hand, poor referral paper indicates that patients were referred with insufficient details which can lead to discontinuity of care, delayed diagnosis, weak follow-up plans, repeated and unnecessary tests, and also inability of the receiving physician to recognize the need for referral, all of which cause reductions in quality of care, medical errors, and increases in health sector expenses.

Moreover, we found that receiving referral feedback was associated with appropriateness of referral system, a finding in line with a recent finding in Ethiopia (22). A study conducted in Iran also stated that the process of referral and patient follow up is disturbed by lack of feedback on the referral system (25). Barriers to feedback provision in the maternal referral system in Ethiopia include ineffective communication channels between health facilities, which hinder timely information exchange. Resource limitations, such as insufficient staff and technology, impede the ability of healthcare providers to offer comprehensive feedback. Additionally, high patient loads often lead to prioritization of immediate care over administrative tasks like feedback provision. Cultural attitudes towards maternal health may also discourage open communication among health workers. Lastly, a lack of accountability and incentives for providing feedback further complicates the referral process (26).

Poor communication among referral staff was strongly reported by all healthcare workers as a major challenge affecting the referral process during obstetric emergencies. These findings are consistent with qualitative study in Ghana (27, 28), and a systematic review in India (29, 30) where access, uptake and effective use of information technology such as electronic health records remain a challenge. These challenges impede effective communication between referral and recipient health facilities. Similar findings in other low resource settings (31) also observed lack of coordination and feedback during referral system and the major challenge in implementation of effective emergency obstetric care. Non-adoption of a responsive and robust communication system in an increasingly complex health system may continue to have implications on quality of care (32), team cohesion and possibly create conflict due to miscommunication (33).

In this high maternal mortality settings, where high delays in seeking care (delay 1) or reaching appropriate facilities (delay 2) and delay in receiving appropriate care (delay 3) including during referral between facilities—improving maternal survival requires strengthening the referral system as these are occurring after reaching a certain level of health care. The cross-sectional nature of this study limits the setting a causal-effect relationship between dependent and independent variables.

## Conclusions

The aim of this study was assessment appropriateness of maternal referral system and associated factors in in Eastern Ethiopia. Only one in ten maternal referrals from public health facilities in eastern Ethiopia were found to be appropriate. Lack of using standard referral forms and referrals during non-working times or days were associated with inappropriate referral process. This study highlights the need for policy makers and healthcare managers to understand the complexity of factors influencing the efficient and effective emergency obstetric referral system. Co-creating standard referral forms with clear elements is essential for making all maternal referrals appropriate and informative. Arranged periodic training for health professionals on effective communication, including feedback and utilization of standard referral form and documentation, would improve referral process thereby the survival of the woman. Further studies might explore the potential impact of referral process issues on maternal and fetal outcomes in low resource settings.

## Data Availability

The original contributions presented in the study are included in the article/Supplementary Material, further inquiries can be directed to the corresponding author.
